# Validation of finite-element simulations with synchrotron radiography – A descriptive study of micromechanics in two-piece dental implants

**DOI:** 10.1016/j.heliyon.2018.e00524

**Published:** 2018-02-08

**Authors:** Wolfram Wiest, Alexander Rack, Simon Zabler, Alex Schaer, Michael Swain, Katja Nelson

**Affiliations:** a Chair for X-Ray Microscopy, University Würzburg, Josef-Martin Weg 63, 97074 Würzburg, Germany; b The European Synchrotron, CS 40220, 38043 Grenoble Cedex 9, France; c Oral Reconstruction Foundation, Margarethenstrasse 38, 4053 Basel, Switzerland; d Biomaterials Science, University of Sydney, NSW 2006, Australia; e Dept. of Oral- and Maxillofacial Surgery, Universityclinic Freiburg, Freiburg, Germany

**Keywords:** Dentistry, Mechanical engineering, Materials science

## Abstract

State-of-the art, two-piece dental implants made from titanium alloys exhibit a complex micromechanical behavior under dynamical load. Its understanding, especially the formation of microgaps, is of crucial importance in order to predict and improve the long-term performance of such implants. Microgap formation in a loaded dental implant with a conical implant-abutment connection can be studied and quantified by synchrotron radiography with micrometer accuracy. Due to the high costs and limited access to synchrotron radiation sources, alternative approaches are needed in order to depict the microgap formation. Therefore, synchrotron radiography is used in this article to validate a simple finite element model of an experimental conical implant design. Once validated, the model is in turn employed to systematically study the microgap formation developed in a variety of static load scenarios and the influence of the preload of abutment screw on the microgap formation. The size of the microgap in finite element analysis (FEA) simulations is consistent with that found in *in-vitro* experiments. Furthermore, the FE approach gives access to more information such as the von-Mises stresses. It is found that the influence of the abutment screw preload has only a minor effect on the microgap formation and local stress distribution. The congruence between FE simulations and *in-vitro* measurements at the micrometer scale underlines the validity and relevance of the simple FE method applied to study the micromovement of the abutment and the abutment screw preload in conical implant-abutment connections under load.

## 1 Introduction

Dental implants made from titanium or titanium alloys have been used successfully for many decades for the replacement of human teeth. Today, a two-piece screw implant is the most common, comprising; I. implant body, II. abutment and III. abutment screw, which connects the abutment axially on the implant [1]. The implant-abutment complex with the crown must withstand multi-axial masticatory forces and provide long-term stability. Implants based on conical implant-abutment connection (IAC) have become increasingly popular. These implants have excellent clinical success rates; however the introduction of smaller diameter implants (≤3.5 mm) with thin implant wall thickness (around 0.3 mm), necessitates examination to ensure adequate fatigue resistance. In the past, controversial discussions about the superior performance of such thin walled implants has taken place [2, 3]. Our previous work demonstrated residual deformation and fatigue cracking upon *in-vitro* testing [4, 5]. Off axis loading induced bending of a conical IAC leads to elastic deformation at the implant wall, causing the abutment to tilt (micro-movement) [5]. While conical IACs seem to perform better in fatigue tests, they have lower bending strength outcomes, compared to butt-joint connections [6]. The associated deformation of the abutment during such loading is small, however it may be characterized by using synchrotron-based microtomography and phase-contrast radiography (PCR) [7, 8, 9]. Contrary to previously published reports these initial studies showed microgap formation evident in all IACs regardless of their design [2, 3]. In addition however, certain geometric parameters of two-piece implants, including taper angle, implant wall thickness, screw diameter, manufacturing precision and diameter-length ratio of the IAC were expected to influence the bending fatigue resistance. Finite element analysis (FEA) enables simulation of mechanical response of dental implants, especially when complemented by experimental observations, and thus provide sensitivity outcomes that show the influence of specific design parameters [10, 11, 12]. These include appropriate tightening torque of the abutment screw, which is essential for IAC mechanical stability [11, 13, 14]. For example, Siamos et al. suggest that torque values larger than 30 Ncm may be applied to guarantee the stability of the joint [15]. Ideally, validated FEA can lead up to virtual implant design.

The aim of this study is simply to validate whether FEA based on linearly elastic deformation is sufficient to predict microgap formation in dental implants. It is explicitly noted that this study does not aim to establish an FE model which describes perfectly the mechanical behavior of a two-piece dental implant design. This is the task for a later, continuing work. Again, the model introduced in this work is simply intended to predict the formation of microgaps. Once validated, it can be extended to quantify the influences of force and application angle, as well as screw mounting torque, on the stability of the abutment in a conical IAC. Furthermore, it would reduce the need for complex characterization methods such as PCR, which have only limited availability. Hence, a simple CAD model of an experimental conical implant was developed, virtually loaded and the resulting microgap at the IAC compared to *in vitro* synchrotron-based PCR results. The latter is a unique property of this study, *i.e.* the experimental validation of a model predicting microscopic structures inside a macroscopic, fully opaque object.

## 2 Materials & methods

### 2.1 Implant and experimental set-up

**Fig. 1:**
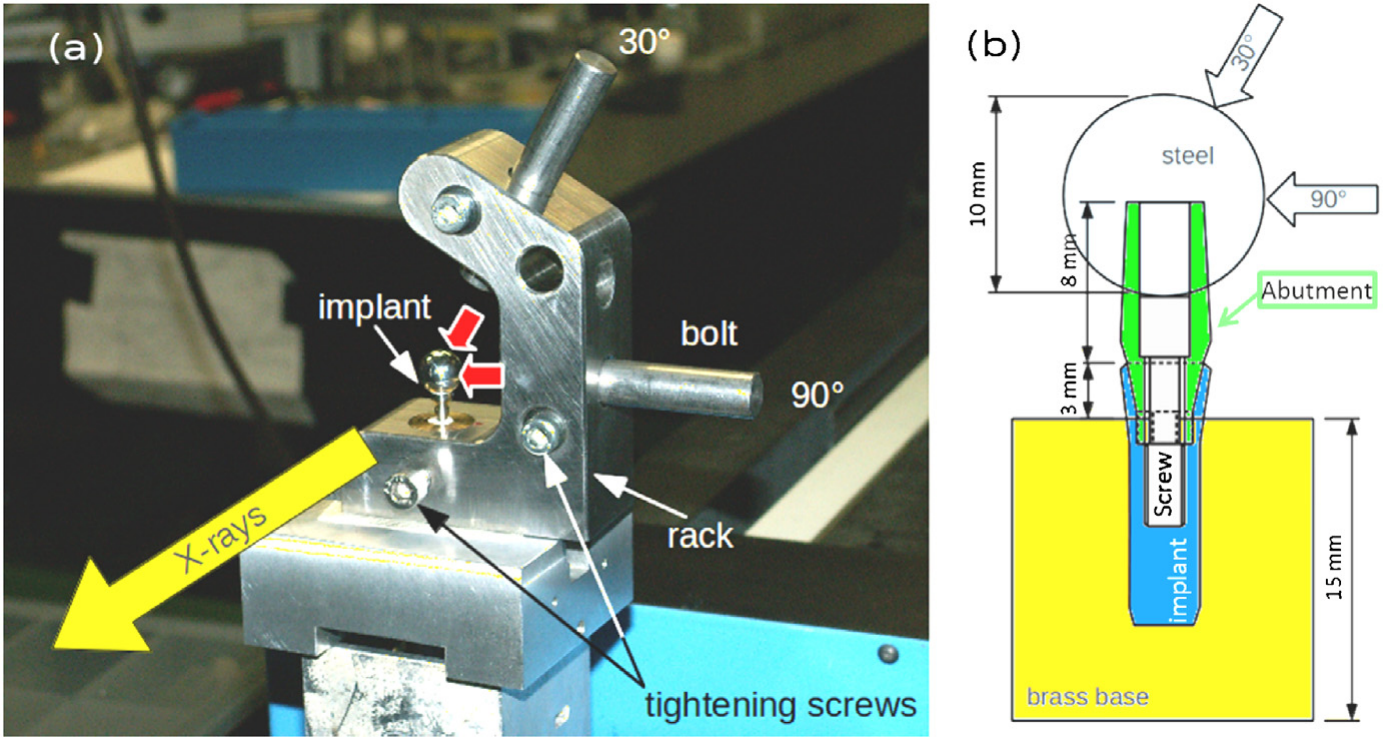
(a) Photo of the testing rig for the Phase-contrast Radiography (PCR) experiments. After load application at 30° or 90°, a bolt sustains the force during the PCR image acquisition. (b) Schematic drawing of the implant assembly according to ISO Standard 14801:2007.

The tested dental implant system is an experimental conical implant (ECI); its geometric parameters are listed in Table 1. The implant body is cp TiGr 4, the screw and abutment of Ti alloy grade 5 (ELI) according to ASTM F-136 and ASTM F-67, respectively. Two implants were tested and IAC microgap measured by synchrotron PCR *in vitro* at the BESSY-II light source. The implant and abutment were assembled and screw tightened with a torque of 15 Ncm. Each implant was rigidly mounted in a brass cylinder (15 × 15 mm^2^) with methylmethacrylate-based adhesive (X60, HBM Germany) up to 3 mm below the shoulder of the implant according to ISO standard 14801:2008 with a 10 mm steel ball connected to the abutment (X60). The center of the steel ball was 8 mm above the shoulder of the implant (cf. Fig. 1b).

**Table 1:** Geometric design parameters according to Fig. 2c of the implant and screw.

Implant system	Taper angle α [°]	Lever angle θ [°]	D_IAC_/L_IAC_ [mm/mm]	D_imp_ [mm]	D_screw_ [mm]	H_index_ [mm]
Experimental Conical Implant (ECI)	7.5	66.2	3.0/1.7	4.3	1.16	0.62

After positioning the implant for PCR, *in-vitro* imaging [9], with a static force of 30, 100 and 200 N at either 90° or 30° to the main implant axis was applied (cf. Fig. 1a). The force was measured with a force gauge (SH-500, PCE-group OHG, Germany), while displacement and IAC microgap were recorded for three static loadings by PCR: 30N-90°, 100N-90° and 200N-30°.

**Fig. 2:**
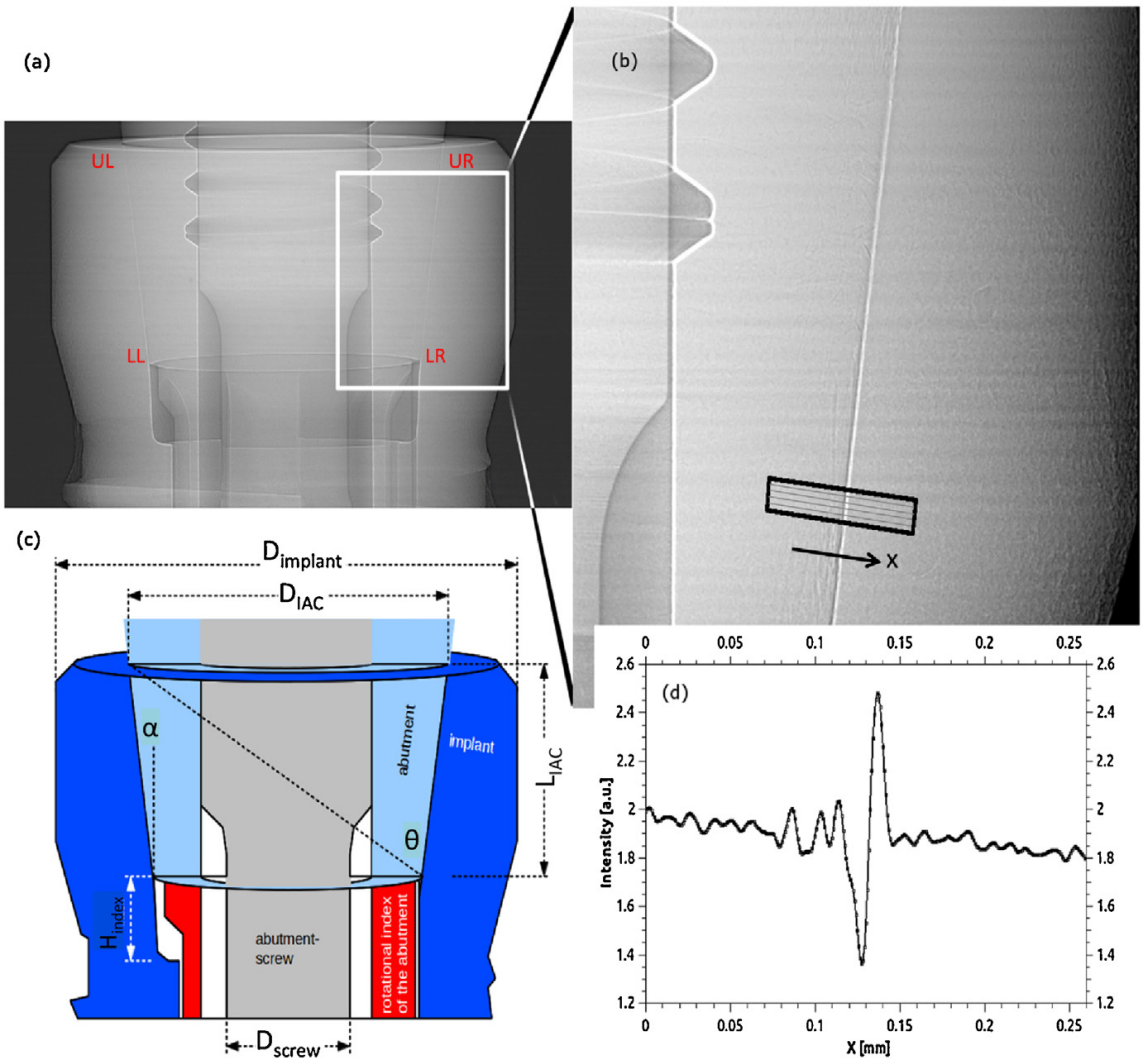
(a) Typical radiograph showing the IAC of the ECI system. (b) Magnified high resolution image in PCR showing the IAC (recorded for 100 N load, at 90° application) and the microgap, which is highlighted by the edge-enhancement effect. (c) Schematic drawing based on (a) showing the different parts and geometric parameters of the ECI implant system. Typical line profiles as in (d) are calculated from strips, which are drawn perpendicular to the IAC, as shown in (b). The line profiles are the basis to calculate the microgap’s width from the PCR [7].

PCR images were recorded at the BESSY-II light source (Berlin, Germany) on the BAMline (with 24.8 pm, X-ray wavelength and 1.4 mm beam-height). The X-ray images were recorded as described previously [16] with 0.87 μm detector pixel size with the detector situated 770 mm downstream of the sample, allowing PCR with substantial edge-enhancement. The IAC microgap is measured by drawing line profiles across the edge-enhanced IAC in the PCR images, see Fig. 2. Calculating the actual microgap width from these profiles is described in an established protocol [7]: using the existing knowledge about the geometry and materials of the implants, the profiles can also be simulated. Here, the gap width is introduced as fit parameter in order to match the simulated profiles to the measured data. A good fit is interpreted as estimator for the width of the microgap [7].

### 2.2 Finite element analysis

**Fig. 3:**
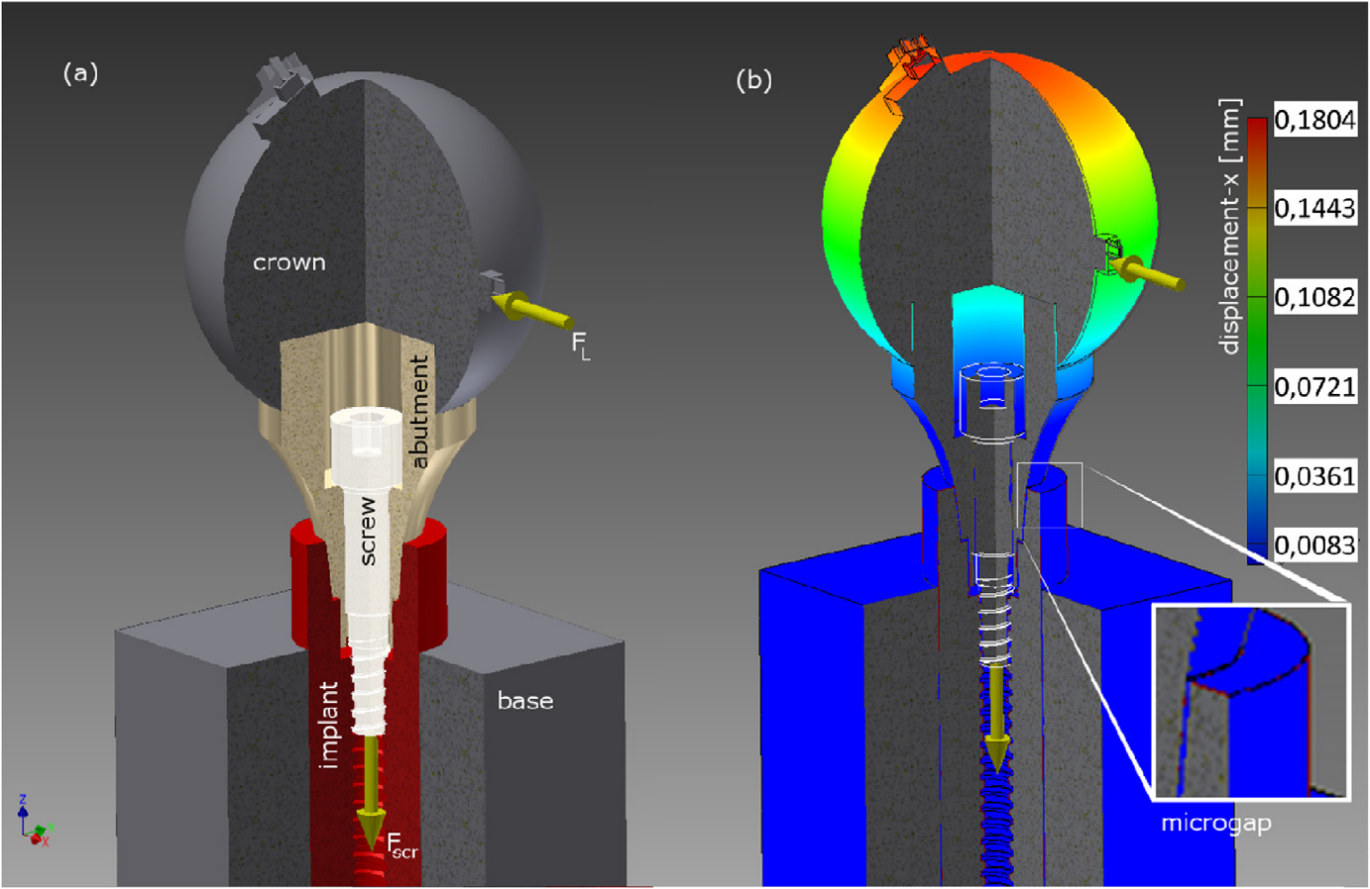
Illustration of the FE simulation for the ECI implant system. (a) The screw assembly preload is simulated by a 120 N downward force, whereas the off-axis load applies to small contacts on the surface of the steel ball in order to guarantee a constant loading angle (either 30°, 45° or 90°). (b) Resulting displacement at 100 N load applied under 90°. The inset shows the precise location in the FE model at the implant shoulder where the microgap is measured (in this case the virtual gap was 9.3 μm wide).

Finite element analysis calculation employed Autodesk Inventor^®^ 3D (Autodesk GmbH, Germany). Based on radiographic observations of the inner and outer geometry, a CAD model was created for the ECI implant as detailed in Fig. 3. The materials’ mechanical parameters used for the simulation are listed in Table 2. The FE model has a number of approximations, namely:

a the abutment screw mounting force F_scr_ is not simulated by a torque, but by a downward force (assembly preload) of the screw (applied at the lower end of the screw, see Fig. 3a).
b The implant is embedded in a solid brass block, no deformation of the block nor movement at the brass-implant interface is assumed in the simulation.
c The same constrain (as in (b)) applies to the interface between steel ball and abutment.

**Table 2:** Mechanical parameters for the FE simulation according to ASTM F-136 and F-67.

Material	Young’s modulus [GPa]	Shear modulus [GPa]	Poisson Ratio	Coefficient of friction	Yield Strength [MPa]
CP-Ti Grade 4	105	40	0.37	0.5	483
Ti Grade 5 ELI	114	44	0.342	0.5	795

Since the screw mounting force is approximated by an elastic downward force the value for the assembly preload F_M_ was estimated from Eq. (1) [17]:

(1) $$F_{M}=M_{A}\cdot {\left(0.16\cdot P+0.58\cdot d_{2}\cdot \mu_{g}+\cos\;\alpha \cdot \frac{D_{Km}}{2}\cdot \mu_{K}\right)}^{-1}$$

with the total tightening torque M_A_ = 15 Ncm, d_2_ = d_1_ − 0.64952 · P = (1.5–0.23) mm = 1.27 mm the pitch diameter, thread pitch P = 0.35 mm, and D_Km_ the average of outer and inner diameters of the friction ring between screw-head and abutment D_Km_ = (d_W_ + D_Ki_)/2 = 1.95 mm. The last term is multiplied with a cosine to account for a tapered screw head in the ECI (α = 32.5°). The friction coefficients μ_G_ (thread) and μ_K_ (head) were both set to 0.5 [18] (own unpublished experiments have never shown values below 0.5). In summary these values result in a preload of the implant system of 179.54 N. Because the torque is different for each implant system from different companies and since the preload has little effect on the microgap formation (see results below), a slightly lower value was used for most of the FE calculations F_M_ = const. = 120 N (which corresponds to 10 Ncm torque) [24, 25].

Standard settings of the Autodesk Inventor^®^ 3D software were applied in order to obtain a generic FE approach that enables estimation of the microgap formation. According to the software description, all of the mesh elements are solid tetra 10 type (4 physical points and 10 nodes for the interpolation). These are volumetric elements with the mesh size at the implant shoulder set to 0.1 mm. At less critical positions the meshes were reduced up to 0.3 mm element size. The FE analysis was calculated with approximately 151 000 nodes and 96 000 elements. The contact condition of the different implant components were defined to be separated [24, 25] (in detail: brass cylinder and implant: bonded, abutment and steel ball: bonded (cf. sample description in Section 2.1), implant and abutment as well as implant and screw: separation and no sliding contact).

A constant force vector was virtually applied to the outer surface of the steel ball at inclinations 30°, 45° and 90° to the main implant axis, in order to simulate the flat surface of the loading bolt which was used during the experiments (cf. Fig. 1a). The width of the microgaps were acquired in the coronal part of the implant-abutment-interface, on the side of force application (named UL = “upper left” in the results) (cf. inset in Fig. 3b). Values of von-Mises stress were attained from the opposite side (termed “point P” for the FEA, or UR = “upper right” for the experimental data). The influence of the screw mounting force, ranging from 0 N to 200 N, on the microgap formation is simulated using a static load of 100 N at an angle of 30°.

It is known that FE simulations are sensitive to the mesh density used [23]. The values chosen above are based on our previous experiences with FE simulations and dental implants [24, 25]. Additionally, simulations were performed with different mesh densities in order to verify that the results are free from any potential influence of the latter (besides the chosen values, simulations were performed for: a) shoulder 0.1 mm, other: 0.2 mm, b) shoulder 0.08 mm, other: 0.150 mm and c) shoulder 0.05 mm, other: 0.08 mm).

## 3 Results

**Fig. 4:**
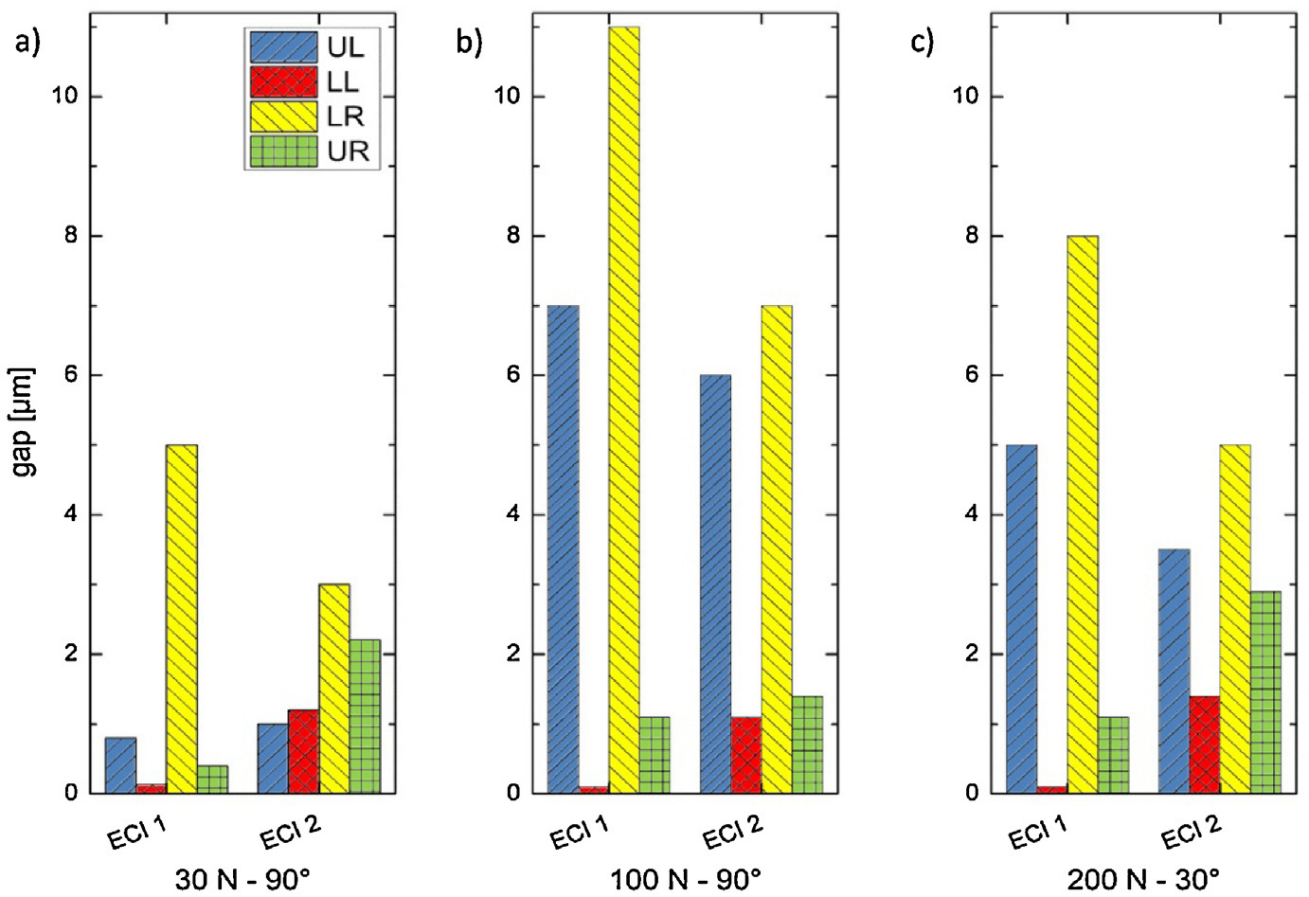
Microgaps calculated from PCR images at the four pivotal points of the tapered IAC (legend: UL = upper left, LL = lower left, LR = lower right, UR = upper right). The applied off-axis load points to the LR direction, therefore a microgap forms at UL and/or LR while a forced contact appears at LL and/or UR. Values are given for three specific loads: (a) 30 N under 90°, (b) 100 N under 90° and (c) 200 N under 30°, inclination with respect to the main implant axis.

The microgap width was determined (Fig. 2) using *in vitro* PCR images and calculating line plots at four different positions at the IAC, labeled “upper left“ (UL), “lower left“ (LL), “lower right“ (LR) and “upper right“ (UR), corresponding to the four edges of the IAC trapezoid (cf. Fig. 2a). Note that the line profiles were calculated approx. 100 μm below and above the exact edges for accuracy. The loading force was always applied from the left hand side, hence point P corresponds to UR and the FE estimations of the microgap have to be compared to UL. Fig. 4 shows the resulting microgap widths. The implants show the largest gap of 11 μm at LR for ECI 1 (at 100 N-90°) and 8 μm for ECI 2 (at 200 N-30°), also in LR. In all load applications the implants open at UL and LR while closing at LL and UR, hence the abutment is canted within the IAC.

**Fig. 5:**
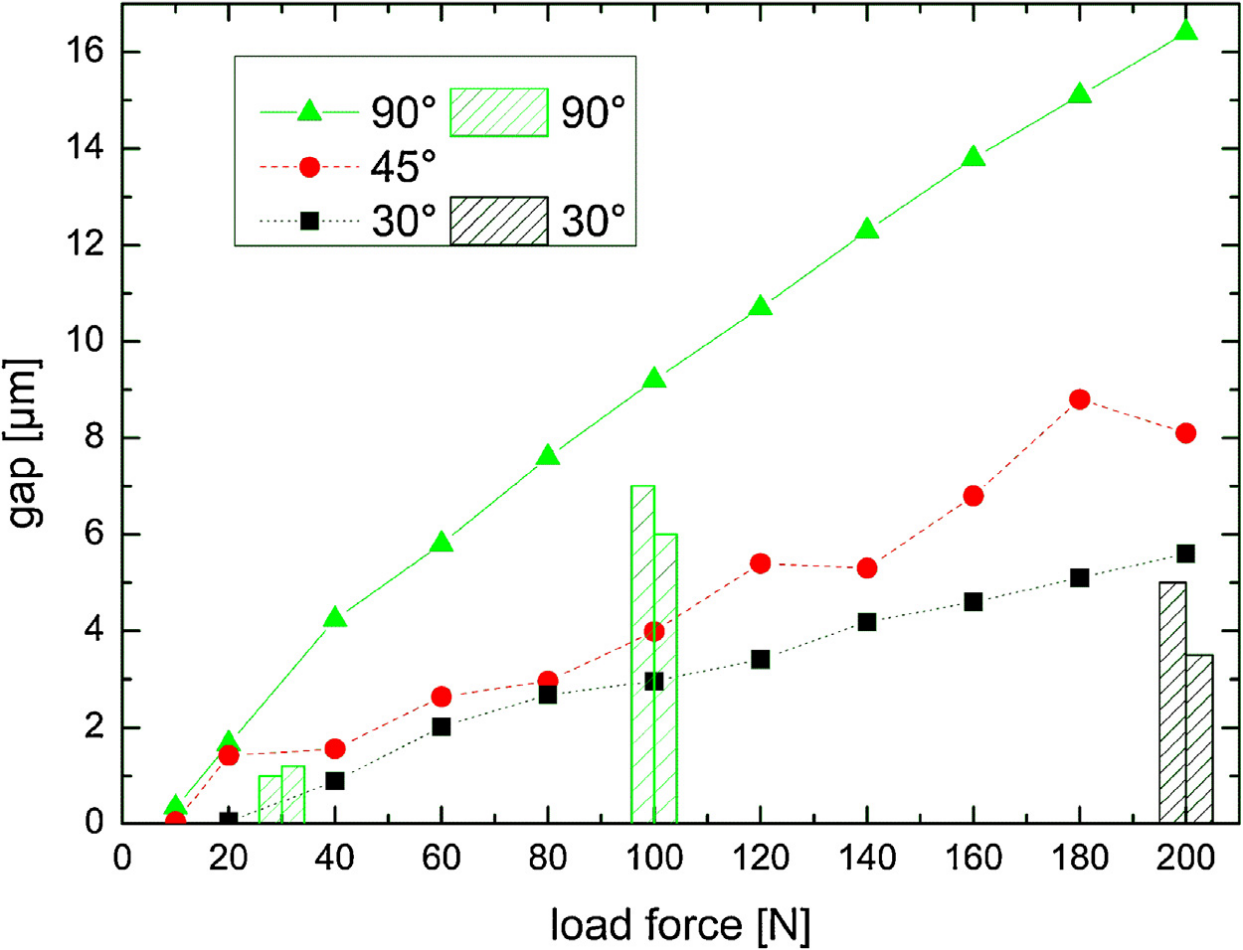
Results of the FE model on the ECI implant: Microgap opening vs. load (ranging from 0 N to 200 N) application at 30°, 45° and 90°. Superimposed onto the FEA results are bars corresponding to the PCR measurements of the microgap in the two tested implants.

**Fig. 6:**
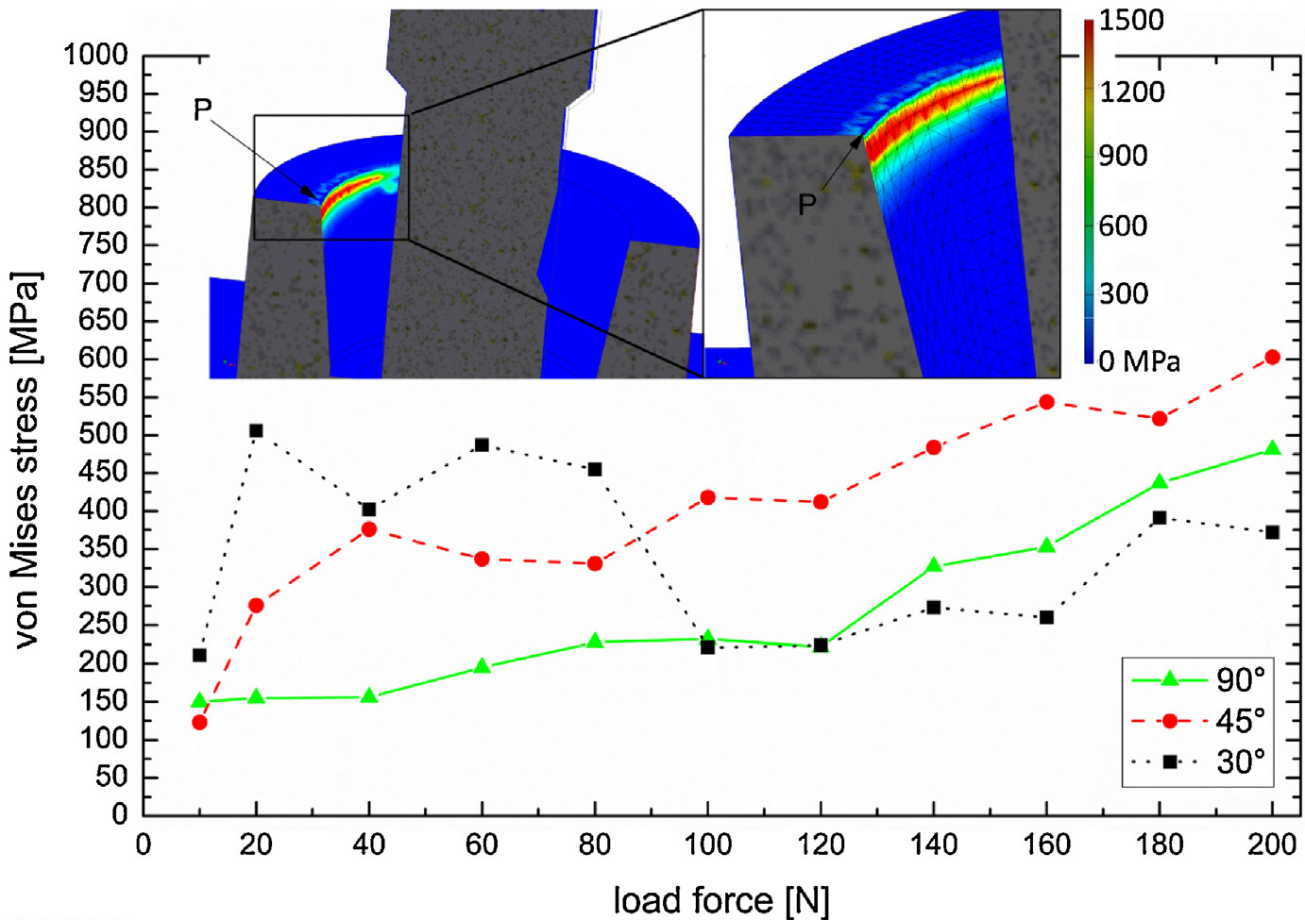
von-Mises stress for F_scr_ = const = 120 N and for 30°, 45° and 90° off-axis force application. Stress values were measured at the inner implant shoulder, one at the pivotal point P. The insets show FE model with the abutment rendered transparent, color values indicating the stress magnitude. The implant shoulder has a mesh size of 0.1 mm.

For the FE model of the ECI implant, microgaps were calculated for axial forces ranging from 10 N to 200 N and angles of 90°, 45° and 30°. The resulting gap widths are shown in Fig. 5, along with the corresponding experimental values (UL in Fig. 4). Obviously, with increasing angle of the applied load the resulting microgaps forming at the adjacent implant shoulder widen, reaching 16.4 μm at 200 N force at 90°, whereas with the same load the gap is only 8.0 μm and 5.7 μm for angles of 45° and 30°, respectively. For each force application angle the IAC microgap width increases monotonically with applied load, but deviates somewhat from linear behavior. Especially at smaller force values (below 50 N, 90° force application) the gap decreases reaching almost zero at 10 N. The data scatter is less pronounced at 90°, it is difficult to estimate the error for both the microgap and stresses from the FE simulations as accuracy improves with a finer mesh at the appropriate locations. Comparing FEA with PCR values the following can be summarized: For 30 N-90° simulation predicted a 3 μm wide gap at UL, while 1.0 μm and 1.2 μm are observed experimentally for samples ECI 1 and 2, respectively. A 9 μm wide gap was expected for 100 N-90° from our simulations, yet we found 7 μm and 6 μm for this configuration. Finally, for 200 N-30° force application 5 μm and 3.5 μm were observed, whereas 5.7 μm was found in the FE study, hence all experimental values are close but 1–3 μm less than of the model. Due to the finite mesh size, von-Mises stresses measured at the opposite implant shoulder (measurement point “P” is indicated in inset, Fig. 6) also exhibit scatter, with peak values as high as 600 MPa (200 N at 45°), which exceeds the yield stress of the CP Ti Grade 4 (483 MPa) implying plastic deformation occurs at the inner implant walls. For the application angle of 45° and 90° von-Mises stress increase monotonically with applied load, whereas high values (>400 MPa) are observed for 30° load application at forces from 20 N to 80 N (Fig. 6). Both the residual stress values for zero load (ca. 150 MPa at 10 N) and fluctuation at 30° application are attributed to the abutment screw preload (120 N for all FE tests).

**Fig. 7:**
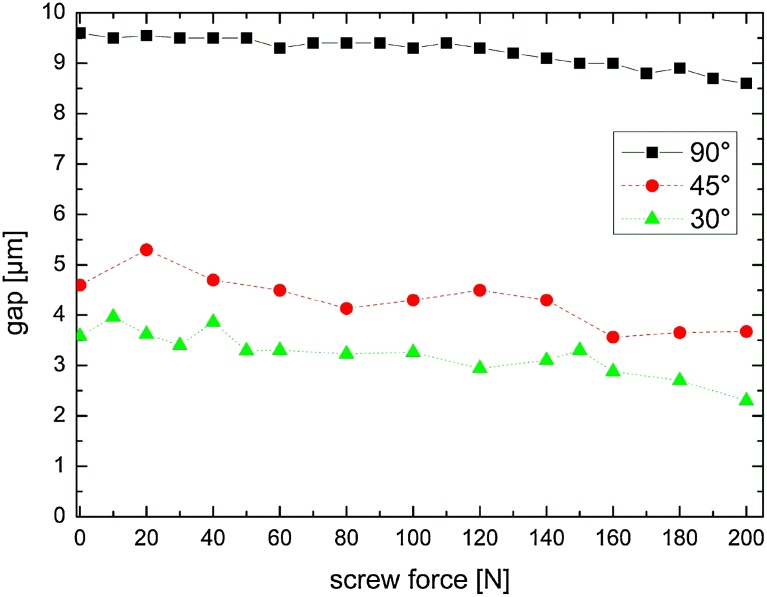
Opening of the IAC microgap as a function of the mounting force of the abutment screw (from 0 N to 200 N) for a constant load of 100 N under 30°, 45° and 90° application angle.

The influence the screw mounting force on the microgap formation at a static load application of 100 N at 30°, 45° and 90° results in microgap widths shown in Fig. 7. The largest gap formation is seen for zero preload (0 N) in all three load directions: 9.6 μm at 90°, 4.5 μm at 45° and 3.6 μm at 30° application. All three values are found to decrease monotonically with the applied preload. This effect is indeed very faint and when the von-Mises stress is examined in the same context (data not shown), no systematic changes in stress as a function of the applied preload can be observed (only stochastic changes).

## 4 Discussion

The FE simulations, based on a simplified linearly elastic model, reveal a high congruence with the experimental microgap values found by *in-vitro* synchrotron-based PCR. Although the simulated microgaps at maximum load are approx. 1–3 μm wider than those measured experimentally. This discrepancy can be attributed to the line profiles being measured approx. 100 μm below or above the IAC extremities. The FE simulation on the other hand enables measurement of microgaps at the implant shoulder level where it shows a greater opening than 100 μm below the shoulder. Hence, the FE model is a useful approach. More sophisticated methods for high resolution spatial measurements, such as the recently developed stroboscopic 4D microtomography of dental implants, are only required in a final stage of the implant development [22].

The implant loading parameters used within this study are based on the ISO Standard 14801:2007 with the implant being embedded in a brass cylinder up to 3 mm below the nominal bone level of the implant. Unfortunately this does not resemble a clinical setting in which the implant is placed in bone with variable quality. Hence, no output can be specifically extracted to conclude the influence of the stress development on the surrounding bone. However, as a consequence of this study the FE model could be modified to include the surrounding bone matrix. Our validation of the FE simulations by experimental measurements of the IAC microgap suggests that such simulations would allow a comparison to be quantified and visualized for *in-vivo* response of loaded implants. Upon refining the model it became apparent that the preload of the abutment screw was an essential parameter for comparison with experimental results. The screw retains the abutment deflection when larger bending forces are applied; hence limiting microgap formation, to an extent, while partially supporting the applied load by straining the threads of the abutment screw. Furthermore, the contact condition of implant and abutment is treated numerically as no sliding contact in our simplified model. It can be expected that the microgap formation is sensitive to the contact condition of interface between implant and abutment.

Once validated these FE models can be used for sensitivity studies to evaluate specific design parameters, *e.g.* IAC taper angle, or implant wall thickness. From the FE model we found that the preload or screw mounting force has limited influence on microgap formation and peak stresses at point P. In fact the screw mounting force is a matter of controversy and a hypothesis exists that with increasing screw preload a decrease in micromovement of the abutment results [15]. These authors indicated mechanical stability of the IAC is correlated to the preload or tensile force generated in an abutment screw by tightening it [15, 19]; this clamping force exerted by the screw must exceed the forces acting on the joint. Micromovements of the components of a joint are known to minimize the preload as seen in samples measured after fatigue loading [15, 20]. The optimum preload should ensure that with additional occlusal loading forces the materials remain within their elastic range. That is, the resultant stresses generated are below the yield stress of the material used. The accuracy of the preload has been determined to be essential for studying the dynamic loading response of implant components [11]. Evaluating the influence of the screw assembly force on the formation of microgaps and the stress distribution at point P with the FE model under oblique force application (30°) however revealed diverging outcomes. For small assembly forces (lower effective bending moment) the microgap decreases due to a tighter fit, whereas the gap is further opened when the assembly force surpasses a critical value (approx. 100 N for 30°–100 N). Evaluation of the von-Mises stress beyond this value has minimal significance because the implant and abutment are no longer in contact at point P. For lower assembly forces and 90° force application the von-Mises stress ranges between 500 and 800 MPa. The largest values, which are seen for 200 N-90°, indicate that plastic deformation of the inner implant wall may take place at point P when strong oblique chewing forces are applied.

To extrapolate our FE model findings to consider the influence the screw mounting torque on the overall mechanics of the implant system, a more refined model of the ECI is recommended. While for the purpose of simulating the microgap formation the FE model proved to be sufficient, however while an estimate of the von-Mises stresses in the implant was possible it was considered to be at the critical limit of the details of the CAD mesh.

The experimental results support the use of FE modeling which is already widely used to assess the biomechanical behavior of dental implants [21, 22]. So far, the results only consider static off-axis loading of virgin implant systems, and can thus not predict cyclic damage caused by fatigue loading. However, *ex-situ* synchrotron micro-CT measurements [8] revealed residual deformation of the implant body at point P in the high-cycle fatigue regime. These observations are in agreement with von-Mises stresses predicted at this point in the current model. Since the microgap formation as well as any other potential cause (*e.g.* local plastic deformation) for loosening of the abutment screw is a cause for clinical revision/intervention, the interest and necessity for studying the mechanics of dental implants will most likely increase with the complexity of their design.

## 5 Conclusion

FE analysis has been successfully validated using *in-vitro* synchrotron radiography. Compared to the use of laboratory X-ray sources the use of synchrotron radiation allows one to probe the interior of the implant with high spatial resolution [5, 26]. As such this is one of the very few validated dental implant FE studies available. Hence, simple linearly elastic FE analysis enables the investigation of abutment micromovement in a conical implant-abutment connection when multi-axial static loading is applied. Additionally the impact of other specific parameters such as screw preload can be studied. While the FE analysis used in our study was able to predict displacements, *i.e.* it full-filled the needs of our descriptive study, a finer mesh is recommended to reliably determine the stresses generated around stress concentration sites such as threads. A more detailed mesh sensitivity study would be required in order to allow for the model to predict the behavior of a two-piece implant design in a truly realistic manner.

## Declarations

### Author contribution statement

Wolfram Wiest and Alexander Rack: Conceived and designed the experiments; Performed the experiments; Analyzed and interpreted the data; Wrote the paper.

Simon Zabler: Conceived and designed the experiments; Performed the experiments; Wrote the paper.

Alex Schaer: Analyzed and interpreted the data; Contributed reagents materials, analysis tools or data.

Michael Swain and Katja Nelson: Analyzed and interpreted the data.

### Competing interest statement

Alex Schaer was an employee of Camlog Biotechnologies AG and Katja Nelson receives lecture honoraria from Camlog Biotechnologies AG.

### Funding statement

This work was supported by the German Research Foundation (DFG grant: Ne 1646/ Za 656).

### Additional information

No additional information is available for this paper.
